# Supplementary material for: Exploring community structure in biological networks with random graphs
Source: BMC Bioinformatics. 2014 Jun 25;15:220. doi: 10.1186/1471-2105-15-220 (PMC4094994; doi:10.1186/1471-2105-15-220)
Supplement: Additional file 1 — Supplementary analysis. Additional analysis of algorithm with figures. [file 1471-2105-15-220-S1.pdf]

Supplementary Analysis

Expected modularity

The strength of community structure in a network with K partitions is defined as

$$Q = \sum_{k=1}^{K} (e_{kk} - a_k^2) \tag{S1}$$

where e_{kk} denotes the fraction of edges within the module k and a_k is the fraction of the total edges of nodes of module k.

Now, if $\overline{d^k}$ is the average-degree of the module k, $\overline{d_w^k}$ is its average within-module degree and s_k is the total number of nodes in the module, then equation S1 can be written as:

$$Q = \sum_{k=1}^{K} \left[\frac{\overline{d_w^k} s_k}{\bar{d} n} - \left(\frac{\overline{d^k} s_k}{\bar{d} n} \right)^2 \right] \tag{S2}$$

where $\bar{d}$ is the average degree of the network and the network size and total modules is n and K respectively. If the average-degree of each module is equal to the average degree of the network, i.e. $\overline{d^1} = \overline{d^2} = ... = \overline{d^K} = \bar{d}$, and if the average within-degree of each module is equal to the average within-degree of the network, i.e. $\overline{d_w^1} = \overline{d_w^2} = ... = \overline{d_w^K} = \overline{d_w}$, then equation (S2) can be written as:

$$Q = \sum_{k=1}^{K} \left[\frac{\overline{d_w} s_k}{\bar{d} n} - \left(\frac{s_k}{n} \right)^2 \right] = \frac{\overline{d_w}}{\bar{d}} - \sum_{k=1}^{K} \left(\frac{s_k}{n} \right)^2 \tag{S3}$$

where $\overline{d_w}$ is the average within-module degree of the network. Now, if all the modules are of equal sizes, equation (S3) can be further reduced to:

$$Q = \frac{\overline{d_w}}{\bar{d}} - \frac{1}{K} \tag{S4}$$

Thus, the expected modularity in this case can be expressed in terms of the ratio of average module degree $\overline{d_w}$ and average total degree $\bar{d}$ of the network, as well as the total number of partitions or modules K in the network.

Tolerance on average-degree and average within-degree of individual modules

We note that Equation S3 can be used to estimate modularity only when module-level average degree and average within-degree match up to the overall network average-degree and average

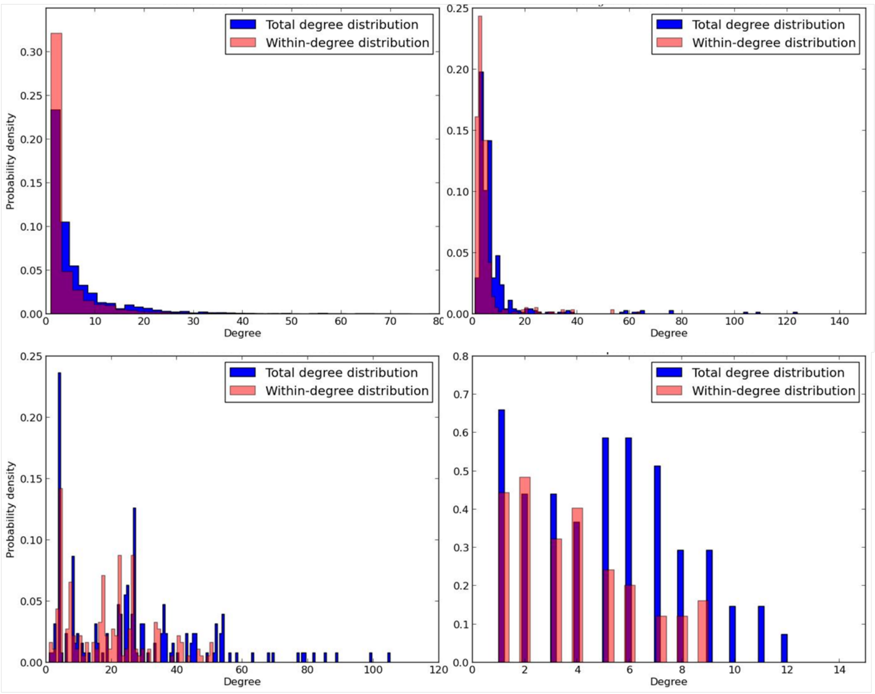

Figure S1: Degree distribution of the four biological networks. The within-degree distribution roughly follows the total degree distribution in all of the networks.

within-degree, i.e., $\overline{d^1} = \overline{d^2} = ... = \overline{d^K} = \overline{d}$ and $\overline{d_w^1} = \overline{d_w^2} = ... = \overline{d_w^K} = \overline{d_w}$. To ensure that these conditions are valid we used rejection sampling of both degree and within-degree sequence. We define the tolerance on the expected modularity, ϵ, to be 0.01 and calculate the tolerance on within-degree and degree sequence as follows:

Let ϵ_{d_w} be the tolerance on sampled within-degree sequence. We define $\epsilon_d = 0.5\epsilon_{d_w}$ to be the tolerance on degree sequence. From Eq. (S2), the observed modularity can be thus written as:

$$\hat{Q} = \sum_{k=1}^{K} \Big[\frac{(\overline{d_w} \pm \epsilon_{d_w}).s_k}{\overline{d}.n} - [\frac{(\overline{d} \pm \epsilon_d).s_k}{\overline{d}.n}]^2 \Big] = \sum_{k=1}^{K} \Big[\frac{\overline{d_w}.s_k}{\overline{d}.n} \pm \frac{\epsilon_{d_w}.s_k}{\overline{d}.n} - \frac{\overline{d}.s_k^2}{\overline{d}^2.n^2} \mp \frac{2.\epsilon_d.\overline{d}.s_k^2}{\overline{d}^2.n^2} \Big] \tag{S5}$$

By ignoring the ϵ_d^2 term which is negligible. $\hat{Q}$ can be further simplified to:

$$\hat{Q} = Q \pm \sum_{k=1}^{K} \Big[\frac{\epsilon_{d_w}.s_k}{\overline{d}.n} - \frac{2.\epsilon_d.\overline{d}.s_k^2}{\overline{d}^2.n^2} \Big] = Q \pm \frac{\epsilon_{d_w}}{\overline{d}} - \sum_{k=1}^{K} \Big[\frac{\epsilon_{d_w}.s_k^2}{\overline{d}.n^2} \Big] \tag{S6}$$

as $\hat{Q} - Q = \epsilon = 0.01$, ϵ_{d_w} can be thus calculated as

$$0.01 = \frac{\epsilon_{d_w}}{\overline{d}} \Big[1 - \sum_{k=1}^{K} \frac{s_k^2}{n^2} \Big] \tag{S7}$$

Within-module degree distribution follows the total degree distribution

In Figure S1 we plotted the probability density of the total-degree and within-degree distribution for four empirical biological networks namely a) Metabolic interaction network of *Caenorhabditis elegans* [1]; b) Food web, depicting the network of trophic interactions at Little Rock Lake in Wisconsin [2]; c) Protein interaction in Yeast [3]; and d) Network of social interactions in a community of 62 dolphins living off Doubtful Sound, New Zealand [4]. We found that the within-degree distribution of most of the empirical networks closely follows the network's total degree distribution indicating a fractal like behavior of the network. Based on this observation we limited our discussions to modular random networks which have similar within-module and total degree distribution. However, our model can be extended to allow for arbitrary within-degree distributions or sequences. To demonstrate, we generated examples of graphs with arbitrary within-degree distributions (Table S1; fourth, fifth and sixth network type) and compared their network properties to modular graphs with similar degree and within-degree distributions (Table S1; first, second and third network type). The modularity value of all generate random graphs was fixed at 0.2. We found that the network properties of clustering coefficient and average path length to be similiar across all the network types (Table S1). Degree assortativity value is close to zero for all network types except for graphs with Poisson degree distribution and geometric within-degree distribution where edge connections are constrained.

Rejection rate of degree and within-degree sequence

Here we estimate the rejection rate of sampling degree and within-degree sequences during the generation of 2000 nodes networks with mean degree 10. The rejection rates are calculated based on the number of times each sequence is rejected per graph generation process. Average rejection rate is calculated over 50 such generation process. Figure S2 shows the expectation of the rejection rate, which we estimate by sampling the average rejection rate ten times. As expected, the rejection rate of sampling degree sequence is similar across the three modularity values. The rejection rate of within-degree sequence increases with network modularity.

Generating disassortative modular random graphs

Anti-modular or disassortative modular random graphs are graphs in which nodes tend to connect to nodes of other modules. This results in within-module edge density to be less than what is expected at random and the value of modularity coefficient, Q, to be negative. In Figure S3 we generate both anti-modular and modular random graphs with identical size ($n = 150$), average degree ($\overline{d} = 5$), number of modules ($K = 3$) and degree distribution (power-law). The absolute Q value of both the graphs is identical (i.e. $|Q| = 0.2$), but anti-modular graph (Fig S3a) has between-module edge density higher than within-module edge density, whereas the opposite is true in the modular random graph (Fig S3b).

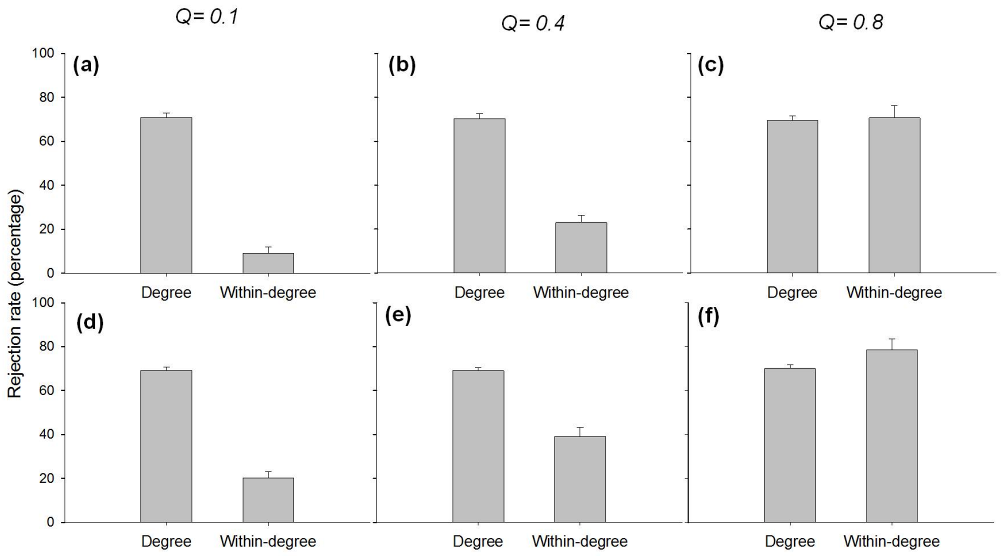

Figure S2: Rejection rate of degree and within-degree sequence sampling for random modular graphs with geometric degree distribution and (a-c) Poisson degree distribution (d-f) Geometric degree distribution. Rejection rate is estimated over three modularity values. The generated graphs have 2000 nodes, mean network degree of 10, and consist of 10 communities.

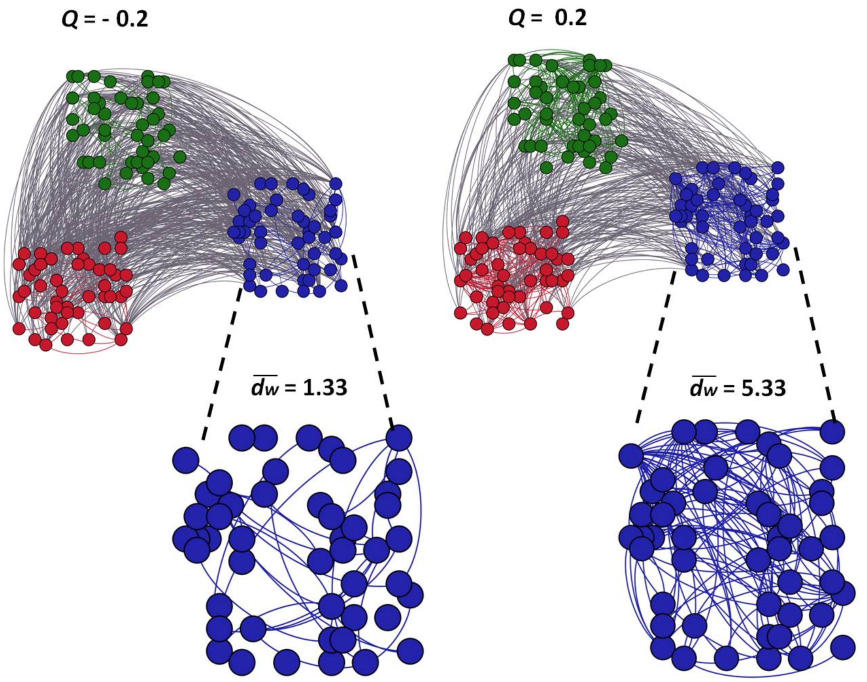

Figure S3: Generating disassortative modular random graphs: Modular random graphs with $n = 150, m = 375, K = 3, P(s = 50) = 1$and p_k is power law with modularity values of : a) Q= -0.2 and b) Q= 0.2. In anti-modular (disassortative) graphs the between-module edge density is more than within-module edge density, whereas the opposite is true in modular random graphs.

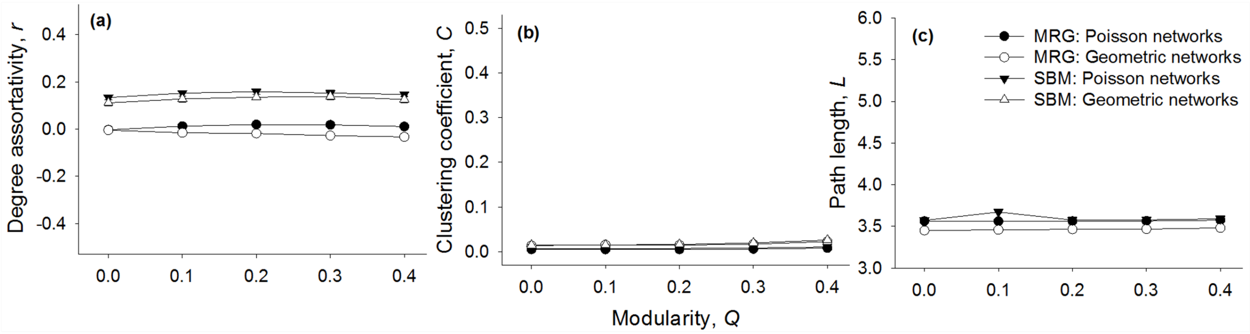

Figure S4: Comparing modular random graphs and stochastic block model (SBM) graphs: Network property of (a) Degree assortativity, (b) Clustering coefficient and (c) Path length in modular random graphs (MRG) and stochastic block model (SBM) graphs of 2000 nodes, mean degree 10 and 10 modules on increasing modularity (Q). Each data point represents the average value of 50 random graphs. The module size of each graph follows a Poisson distribution with mean size of 200. Data points for path length of SBM geometric networks is missing as the generated networks are disconnected. Standard deviations are plotted as error bars.

Comparing structural properties of modular random and SBM graphs

Here we compare the structural properties of modular random graphs generated by our model to the ones generated by degree-corrected stochastic block models (DC-SBM) as described in [5]. SBM is defined by a $k \times k$ stochastic block matrix, where k is the number of modules and M_{ij} gives the probability that a node of module i is connected to a node of module j. The DC-SBM version further defines a propensity parameter γ_u that controls the expected degree of node u.

We used a Python module (*graph-tool*) to generated SBM graphs. Since a formal relationship between the SBM parameters and modularity does not exist, we manually adjusted the parameters values to achieve the desired level of modularity and network parameters. Figure S4 shows two types of SBM graphs: (a) random graphs with Poisson degree-distribution and Poisson within-degree distribution, and (b) random graphs with geometric degree and within-module degree-distribution. Using the Python module and desired network parameters, we were able to generate graphs with a maximum modularity value of 0.4 for both these network types. We therefore generated fifty random graphs at each level modularity and estimated the average values of degree assortativity (Figure S4a), clustering coefficient (Figure S4b) and path length (Figure S4c) of these graphs. The module size follows a Poisson distribution in each of these graphs. To compare DC-SBM to the modular random graphs generated by our model, we generated graphs with identical network parameters and modularity values and report their network properties was well. As Figure S4 shows the structural properties of the graphs generated from the *graph-tool* Python module are similar to those generated by our algorithm. We note, however, that this is a limited comparison and highy dependent on the implmentation of the SBM in *graph-tool.* As discussed in the Previous Work section of the main article, full use of the SBM for generating benchmark or null networks remains to be fully explored.

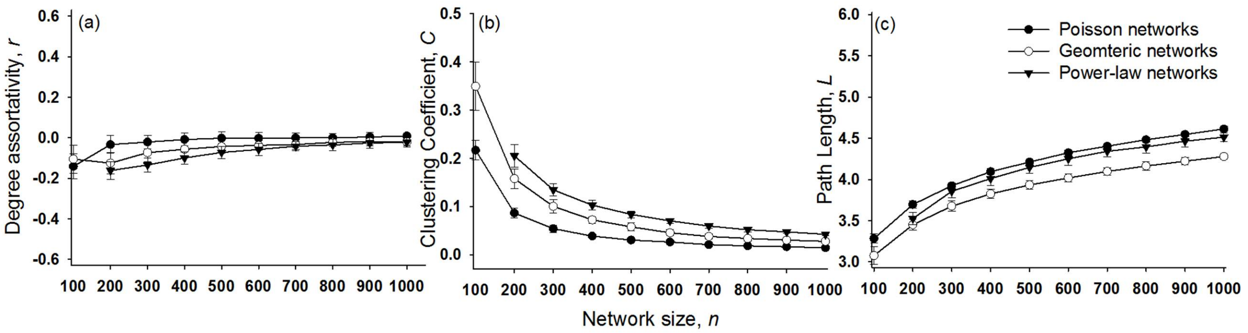

Figure S5: Network property of (a) Degree assortativity, (b) Clustering coefficient and (c) Path length in random modular graphs of mean degree 5 and 10 modules on increasing network size (n). Each data point represents the average value of 50 random graphs. Standard deviations are plotted as error bars.

Effect of network size on network properties of modular random graphs

Here we varied the network size keeping the ratio of community size to the total network size (i.e $\overline{s}/n = 0.1$) constant. As each network comprised 10 communities, increase in total network size also corresponds to the increase in average community size. We observed that, except for very small networks, the assortativity coefficient remains close to zero for all network size (Figure S5a). The negative degree correlation for small networks can be explained by the structural degree cut-off constraint in the communities, i.e. indegree of nodes in a community can attain a value of at-most equal to its community sizes ($max(wd) \leq n_c$). For smaller networks, the highest value of wd is constrained by the small average community size, which results in the total number of high indegree to be much less than expected. Thus, during the randomization step the high indegree nodes connect much more to the low degree nodes which result in disassortative network. A similar observation was noted in hierarchically modular networks by Jing [6]. Clustering coefficient is higher for small networks but decreases to a value close to zero in networks with more than 400 nodes, which is observed in larger networks as well (Figure S5b). As expected, the average shortest path length increases proportionally with network size (Figure S5c).

Effect of average network degree on network properties of modular random graphs

We next tested the effect of network mean degree on other properties of the network. We observed that geometric and power-law null modular networks become disassortative with higher $\overline{d}$ value, while Poisson networks do not show any assortative interaction at any value $\overline{d}$ (Figure S6a). The tendency of geometric and power-law null modular networks to become disassortative could again be due to the structural cut-off constraint of nodal indegrees. As the average degree increases, the graph becomes more dense and hence creates more implicit triangles, resulting in a gradual increase in clustering (Figure S6b). Decrease in average shortest path length with increase in mean network

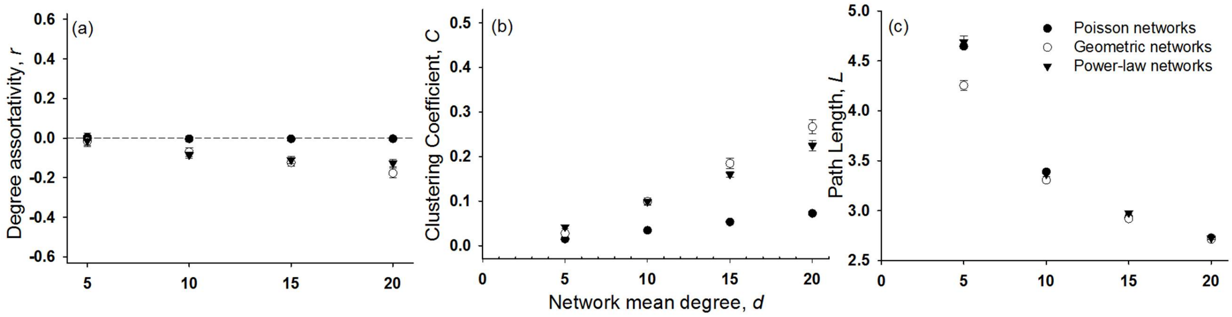

Figure S6: Network property of (a) Degree assortativity, (b) Clustering coefficient and (c) Path length in random modular graphs with 10 modules over a range of mean network degree. Each network has 1000 nodes. The data point represents the average value of 50 random graphs. Standard deviations are plotted as error bars.

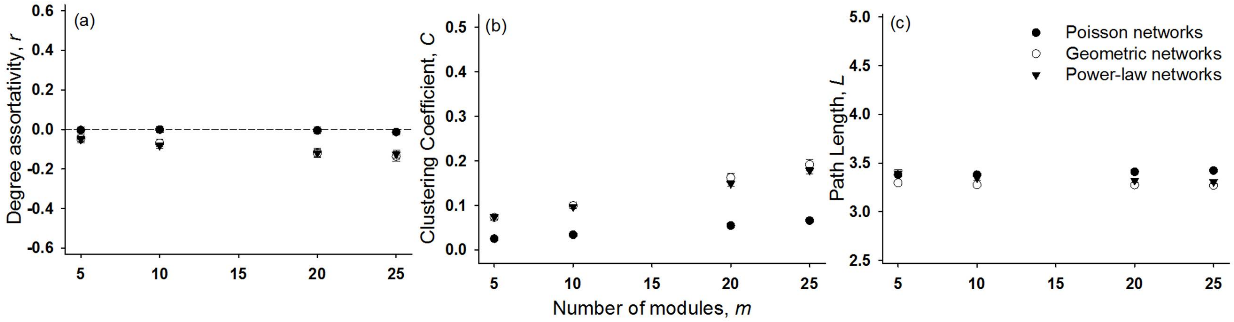

Figure S7: Network property of (a) Degree assortativity, (b) Clustering coefficient and (c) Path length in random modular graphs of size 1000 with mean degree 10 but different number of modules. As the total network size is fixed (=1000) and each module in a network is of equal size, increasing the number of modules in a network corresponds to a decrease in average community size. Each data point represents the average value of 50 random graphs. Standard deviations are plotted as error bars.

degree is also well known [7, 8].

Effect of average community size on network properties of modular random graphs

We also investigated the effect of average community size on the network properties of the null modular network. Figure S7 summarizes the results for networks with a network size of 1000 but different number of modules. A smaller number of modules thus corresponds to a larger average community size. We observed that the community size does not effect the assortative interaction for Poisson networks (Figure S7a). Geometric and power-law networks show disassortative interactions in networks with small community size due to structural degree cut-off constraint explained above. The density of edges within smaller communities is high, which causes high clustering (Figure S7b). However, the average shortest path length is unaffected by the community size (Figure S7c) as the

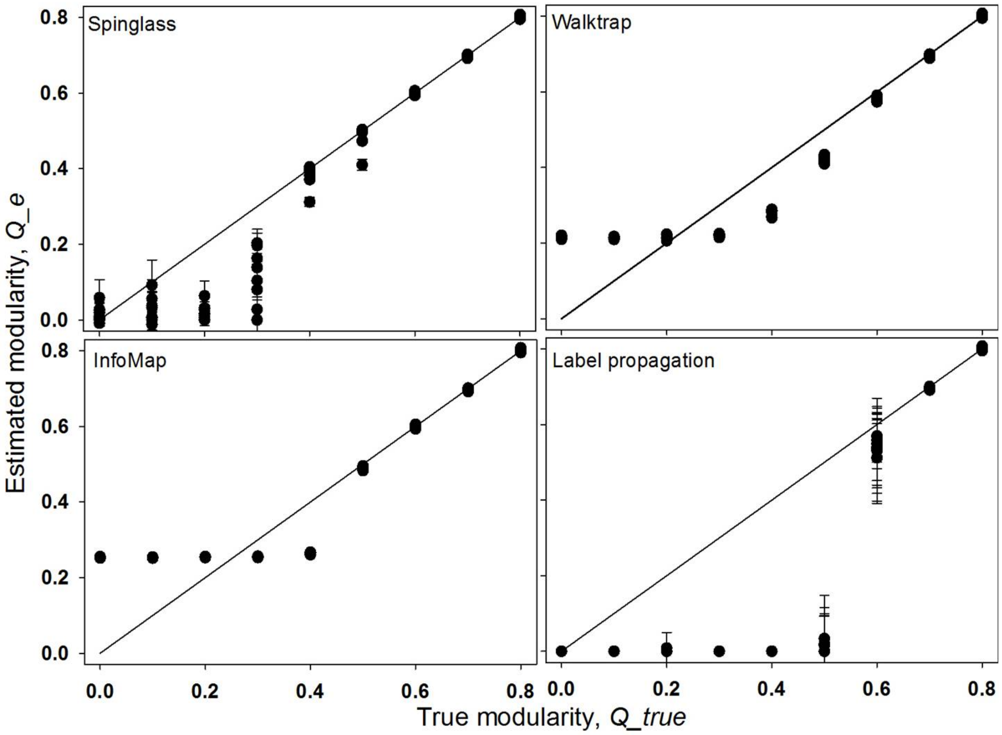

Figure S8: Performance of various community detection algorithms on random modular networks with Poisson degree distribution. Network size n= 2000, mean degree ($\bar{d}$)=10, number of modules (m)=10. Each data point represents the average results of 25 detection runs on a generated modular random network. For each Q value 10 modular random networks were generated.

total network size and network mean degree is constant across all network types.

Performance of other community detection algorithms on modular random graphs

Here we estimated the modularity of our generated random modular Poisson (Figure S8), geometric (Figure S9), and power-law (Figure S10) networks using four additional community detection algorithms namely: (a) Spinglass or Potts model [9]; (b) Walktrap algorithm [10], (c) Infomap algorithm [11], and (d) Label propagation model [12]. Overall, the accuracy of these algorithms improves with increasing Q value.

Accuracy of network partitioning by Lovain and fast modularity algorithm

We tested the accuracy of network partitioning by Louvain and fast modularity algorithm (Figure S11) in random modular networks with a mean network degree of 10 using Jaccard similarity index (J) and variation of information (VI) as a measure of similarity. Jaccard index is the ratio

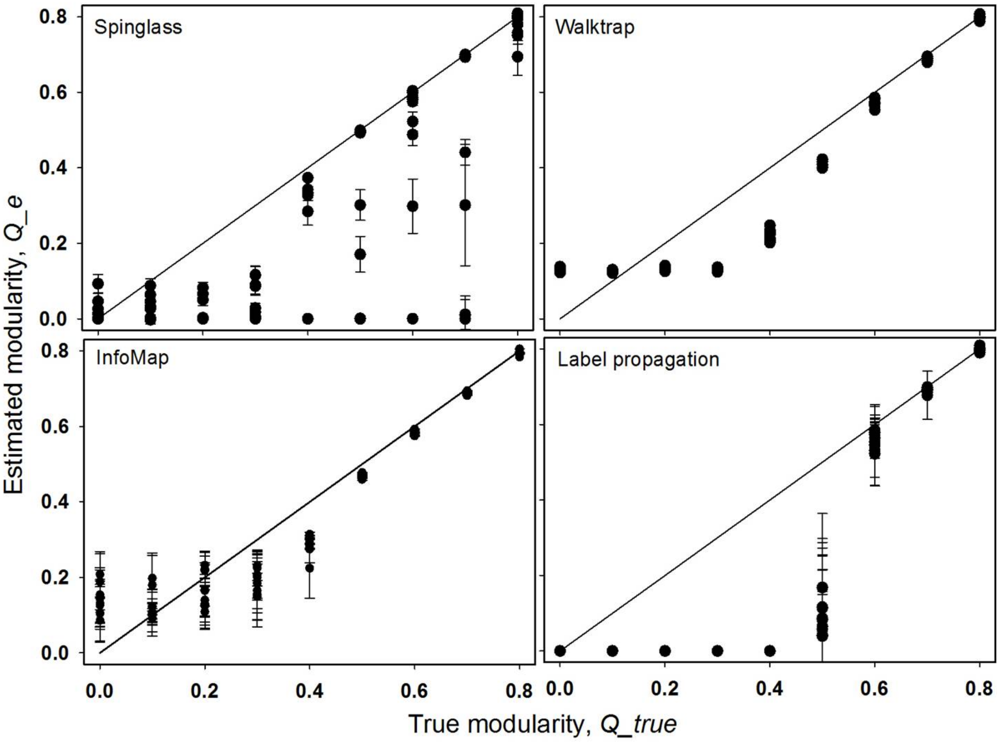

Figure S9: Performance of various community detection algorithms on random modular networks with geometric degree distribution. Network size n= 2000, mean degree $(\bar{d})$=10, number of modules (m)=10. Each data point represents the average results of 25 detection runs on a generated modular random network. For each Q value 10 modular random networks were generated.

.

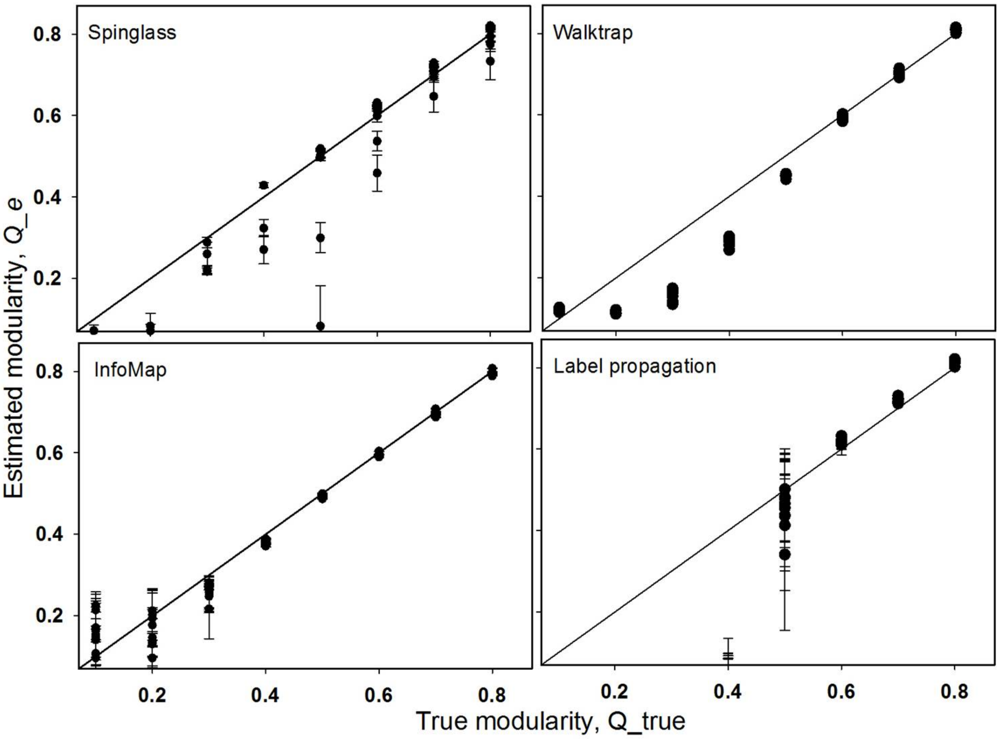

Figure S10: Performance of various community detection algorithms on random modular networks with power-law degree distribution. Network size n= 2000, mean degree $(\bar{d})$ = 10, number of modules (m)=10. Each data point represents the average results of 25 detection runs on a generated modular random network. For each Q value 10 modular random networks were generated.

.

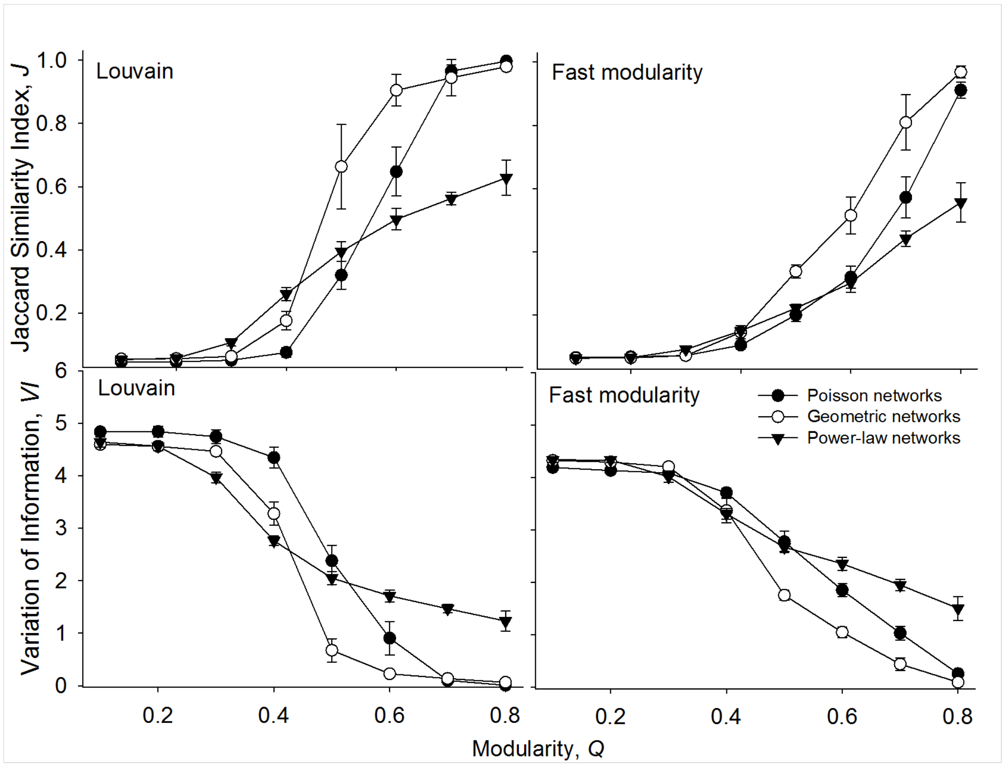

Figure S11: Accuracy of partitions detected by Louvain and fast modularity algorithm in networks with mean degree 10 measured by Jaccard similarity and variation of information index. Fill circles, open circles and triangles represent networks with Poisson, geometric and power-law degree distribution respectively. Each data point represents the average result for ten random networks. Error bars denote standard deviations.

of the number of nodes classified in the same module by both the partitions to the total number of nodal pairs, i.e.

$$J = \frac{w_{11}}{w_{11} + w_{01} + w_{10}} \tag{S8}$$

where w_{11} represent the number of nodal pairs that are in the same module for both the partitions, w_{00} are the nodal pairs that are in different modules in both the partitions and $w_{10}(w_{01})$ are the number of pairs that are put together in the same module by one partition but not by the other. The value of Jaccard index ranges from 0 to 1, with 1 indicating a perfect partition match.

VI measures the amount of information lost and gained in changing from clustering C to clustering C' [13] and is defined as

$$VI(C, C') = H(C|C') + H(C'|C) \tag{S9}$$

or,

$$VI(C, C') = [H(C) - I(C|C')] - [H(C') - I(C|C')] \tag{S10}$$

where $H(C)$ and $H(C')$ represents uncertainty in cluster C and C' respectively, and $I(C|C')$ is the mutual information between the two clustering. In other words, the first term of equation (S10) measures the amount of information that we loose, while the second term measures the amount of information that we gain, when going to clustering C' from C.

Null analysis of empirical networks

We generated random modular graphs for each of the four biological networks by randomizing the within-edge and between-edge connections. Specifically, we generated 50 such random graphs using the estimates of total degree distribution, within-degree distribution, and distribution of module size $P(\bar{s})$ as the empirical network but used our model to connect the within- and between- edges. We next measured networks properties such as clustering (C), average path length (L), assortativity (r) for each of the random network and computed the ensemble mean. Table 1 records the value of each of these properties for the empirical networks and the relative deviation of the ensemble mean of random modular graphs from the observed value (i.e. deviation = [observed value - ensemble mean/observed value])

Table S1: Comparisons of random modular networks with different degree- and within-degree distributions type.

Network types	Modularity, Q	Degree assortativity, r	Clustering coefficient, C	Path length L
1) Poisson degree distribution, Poisson within-degree distribution	0.2 [0.004]	0.02 [0.009]	0.01 [0]	3.56 [0.002]
2) Geometric degree distribution, Geometric within-degree distribution	0.2 [0.003]	-0.03 [0.009]	0.02 [0.001]	3.48 [0.010]
3) Power-law degree distribution, Power-law within-degree distribution	0.2 [0.002]	-0.01 [0.009]	0.01 [0]	3.48 [0.009]
4) Poisson degree distribution, Geometric within-degree distribution	0.2 [0.003]	0.13 [0.009]	0.007 [0]	3.57 [0.002]
5) Geometric degree distribution, Power-law within-degree distribution	0.2 [0.002]	-0.03 [0.009]	0.02 [0.001]	3.49 [0.013]
6) Geometric degree distribution, Poisson within-degree distribution	0.2 [0.004]	0.03 [0.009]	0.01 [0.001]	3.50 [0.011]

Network property of assortativity, clustering coefficient, and path length in random modular graphs of size 2000 with mean degree 10. Each network type represents random modular graphs with a specific degree and within-degree distribution. Module sizes of all the generated networks follow a Poisson distribution. Each value represents an average of 50 random graphs. Standard deviations are included within square brackets.

Table S2: Comparisons of empirical and random networks with randomized within-edge and between-edge connections

Biological Network Type	N	k	Q	C	L	r
Little Rock Foodweb Interactions	183	26.79	0.36	0.32 [-88%]	2.15[1%]	-0.26[69%]
Yeast Protein Interactions	4713	6.31	0.54	0.09 [-44%]	-	-0.14 [228%]
C.elegans Metabolic Interactions	453	9.01	0.44	0.65 [22%]	2.66 [1%]	-0.22 [0]
Dolphin Social Interaction	62	5.13	0.52	0.26 [-12%]	3.36 [1%]	-0.04 [450%]

For each of the four empirical network we generated 50 null modular network constrained to have the same total-, within- and between-degree list as the empirical network. The table summarizes network statistics of empirical network *viz.* the network size (N), average network degree (k), modularity(Q), clustering (C), average shortest path-length (L) and degree assortitativity (r). The value in brackets is the relative deviation of ensemble mean of null modular networks from the observed value. The path length value for the empirical Yeast-Protein interaction network is missing as the network is not fully-connected

References

[1] H Jeong, B Tombor, R Albert, Z N Oltvai, and a L Barabási. The large-scale organization of metabolic networks. *Nature*, 407(6804):651–4, October 2000.

[2] ND Martinez. Artifacts or attributes? Effects of resolution on the Little Rock Lake food web. *Ecological Monographs*, 61(4):367–392, 1991.

[3] Vittoria Colizza, Alessandro Flammini, Amos Maritan, and Alessandro Vespignani. Characterization and modeling of protein–protein interaction networks. *Physica A: Statistical Mechanics and its Applications*, 352(1):1–27, July 2005.

[4] David Lusseau, Karsten Schneider, Oliver J. Boisseau, Patti Haase, Elisabeth Slooten, and Steve M. Dawson. The bottlenose dolphin community of Doubtful Sound features a large proportion of long-lasting associations. *Behavioral Ecology and Sociobiology*, 54(4):396–405, September 2003.

[5] Brian Karrer and MEJ Newman. Stochastic blockmodels and community structure in networks. *Physical Review E*, 83:1–11, 2011.

[6] Z Jing, T Lin, Y Hong, and L Jian-Hua. The effects of degree correlations on network topologies and robustness. *Chinese ...*, 16(12):3571–3580, 2007.

[7] S. Dorogovtsev, J. Mendes, and J. Oliveira. Degree-dependent intervertex separation in complex networks. *Physical Review E*, 73(5):056122, May 2006.

[8] Janusz Hołyst, Julian Sienkiewicz, Agata Fronczak, Piotr Fronczak, and Krzysztof Suchecki. Universal scaling of distances in complex networks. *Physical Review E*, 72(2):026108, August 2005.

[9] J Reichardt and Stefan Bornholdt. Statistical mechanics of community detection. *Physical Review E*, 74:1–16, 2006.

[10] Pascal Pons and Matthieu Latapy. Computing communities in large networks using random walks. *Journal of Graph Algorithms and Applications*, 10(2):191–218, 2006.

[11] M. Rosvall, D. Axelsson, and C. T. Bergstrom. The map equation. *The European Physical Journal Special Topics*, 178(1):13–23, April 2010.

[12] Usha Raghavan, Réka Albert, and Soundar Kumara. Near linear time algorithm to detect community structures in large-scale networks. *Physical Review E*, 76(3):036106, September 2007.

[13] M Meilă. Comparing clusterings by the variation of information. *Learning theory and kernel machines*, pages 173–187, 2003.
